# ERRATA

**DOI:** 10.1590/1413-785220172504167561ERRATA

**Published:** 2017

In the article entitled “SURVIVAL OF NONAGENARIAN PATIENTS WITH HIP FRACTURES: A COHORT STUDY” authored by Alexa Ovidiu; Gheorghevici Teodor Stefan; Popescu Dragos; Veliceasa Bogdan; Alexa Ioana Dana, published in Revista Acta Ortopédica Brasileira (ACTA) vol. 25 nº 4, 2017, page 132, DOI: http://dx.doi.org/10.1590/1413-785220172504167561, by request of the authors.

The text reading: Citation: Ovidiu A, Stefan GT, Dragos P, Bogdan V, Dana AI. Survival of nonagenarian patients with hip fractures: a cohort study. Acta Ortop Bras. [online]. 2017;25(4):132-6. Available from URL: http://www.scielo.br/aob. 

Is replaced with: Citation: Alexa O, Gheorghevici TS, Popescu D, Veliceasa B, Alexa ID. Survival of nonagenarian patients with hip fractures: a cohort study. Acta Ortop Bras. [online]. 2017;25(4):132-6. Available from URL: http://www.scielo.br/aob. 

